# A Case of Peritoneal Encapsulation Presented as Acute Mechanical Small Bowel Obstruction: A Case Report and a Brief Literature Review

**DOI:** 10.1155/2022/7851130

**Published:** 2022-08-11

**Authors:** Khalil Abuzaina, Ahmad Abuayash, Hidaya Al-Shweiki, Mohammad O. M. Hroub, Anwar Yousef Jabari, Shayma Hafiz, Tuqa Abu Ihlayel, Balqees Mohsen, Sulaiman Naji Fakhouri, Murad Jaa'freh

**Affiliations:** ^1^General Surgery Department-Governmental Hebron Hospital, State of Palestine; ^2^Department of Radiology-Governmental Hebron Hospital, State of Palestine; ^3^Faculty of Medicine-Palestine Polytechnic University, State of Palestine

## Abstract

Peritoneal encapsulation (PE) is a rare congenital malformation in which the small intestine is partially or totally encased in a supplementary peritoneal sac. PE is usually asymptomatic; therefore, it is one of the rarest etiologies of bowel obstruction. Our patient presented at the age of 55 with no prior surgical history and a 3-day history of abdominal pain associated with nausea, vomiting, belching, and constipation. An obstruction secondary to an internal hernia—visualized on a CT scan—was suspected as the initial etiology. On exploratory laparotomy, the small bowel was covered by a thick adherent sac. These findings are consistent with PE, a condition that deserves recognition among clinicians worldwide. Intraoperatively, the sac was excised, and the small bowel was pulled up to the peritoneal cavity starting from the ileocecal valve to the duodenojejunal junction. In the postoperative period, the patient was managed with intravenous fluids, analgesics, and antibiotics. Wound infection was the only postoperative complication. Otherwise, all symptoms subsided, and the patient improved and was discharged home on the 8th postoperative day.

## 1. Introduction

Small bowel obstruction is a typical surgical emergency, which often occurs due to postoperation or obstructed hernia adhesions [1]. One of the rarest etiologies of bowel obstruction is peritoneal encapsulation, a rare congenital malformation. The small intestine is partially or totally encased in a supplementary peritoneal sac [1–3]. Peritoneal encapsulation (PE) is usually asymptomatic. Therefore, it is often diagnosed accidentally, whether during laparotomy or autopsy [1, 3]. Despite that, PE may present with small bowel obstruction in rare cases [1, 3, 4]. In this case report, we describe a case of PE presented as an acute mechanical small bowel obstruction. Furthermore, we do a brief literature review for similar cases of PE that presented with a picture of small bowel obstruction.

## 2. Case Presentation

A 55-year-old male was admitted to the emergency department due to 3-day history of severe colicky left lower quadrant abdominal pain radiating to the back and associated with nausea, bilious vomiting, belching, and constipation. The patient had been having the same symptoms for over 35 years, and he used to relieve them with vomiting. This time, the pain did not subside with vomiting, and he became unable to bear it. The patient is a known case of type 2 diabetes mellitus, hypertension, hyperlipidemia, and hyperthyroidism. He had no past surgical history. He was on metformin for type 2 diabetes mellitus, Bisoprolol for hypertension, atorvastatin for hyperlipidemia, and methimazole for hyperthyroidism. He had no relevant social or family history.

**Figure 1:**
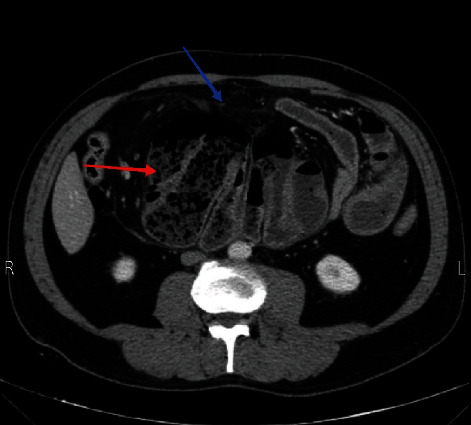
Axial image of contrast enhanced CT shows cluster of small bowel (red arrow) which encapsulated by peritoneum (blue arrow).

**Figure 2:**
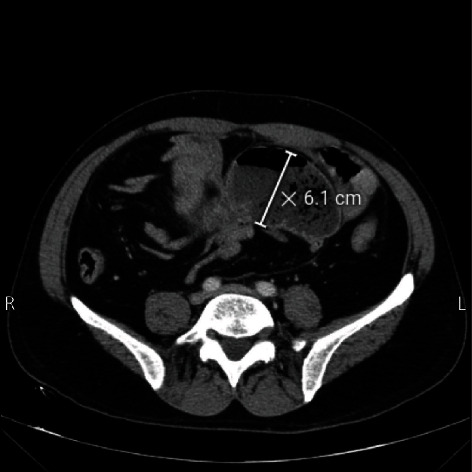
Axial image of contrast-enhanced CT shows dilated small bowel loop, a sign of small bowel obstruction.

On physical examination, the abdomen was tender and rigid. An abdominal CT scan was obtained for further clarification, showing a small bowel obstruction and suspecting an internal hernia (Figures 1 and 2). The patient was admitted to the surgical department in preparation for exploratory laparotomy.

Under general anesthesia, a midline incision was done, and all abdominal wall layers were opened. The caecum was identified, but all small bowel was retroperitoneal and covered by a thick adherent sac. The surgeon started releasing the adhesions to expose the small bowel from the ileocecal valve. Although the adhesions were thick, all small bowel was released up to the duodenojejunal junction, which was dark in color, so warmed normal saline was applied. As a result, the small bowel returned to its normal color with visible peristalsis, and the sac was excise. There were two iatrogenic small bowel perforations repaired in two layers. An appendectomy was done during the adhesiolysis as it was adherent to the small bowel, and the hemostasis was secured. Two abdominal drains were applied, and then, the abdomen was closed. In the postoperative period, the patient was managed with intravenous fluids, analgesics, and antibiotics. Wound infection was the only postoperative complication. Otherwise, all symptoms subsided, and the patient improved and was discharged home on the 8th postoperative day.

## 3. Literature Review

In this section, we summarize the characteristics of a worldwide 25 cases of peritoneal encapsulation that have been published previously. It includes 17 male and eight female patients with a range of ages from 12 to 87 years old. They presented with a picture of acute or chronic small bowel obstruction. Their symptoms include colicky abdominal pain, nausea, anorexia, and vomiting. All of them were treated successfully by surgical resection of the sac (Table 1).

## 4. Discussion

This case report highlights the first described by Cleland in 1868 [5]. It is a very rare congenital malformation with less than 50 cases reported in the literature [6]. As a result of this low number, the etiology is not well understood yet, and most patients are diagnosed accidentally. An accessory peritoneal membrane that covers part of the small bowel is the most likely etiology for this condition [7]. Even though the cause is poorly understood, most of the existing theories suggest that PE occurs probably due to malrotation of the bowel during the 12th fetal week and, as a result, an abnormal return of the midgut into the abdominal cavity of the fetus occurs [2].

During normal fetal development, the yolk's sac coat stays in the umbilical pedicle, while in PE, the coat migrates to the intestine, causing the formation of an accessory peritoneal membrane [2]. PE, however, can occur with other congenital anomalies such as incomplete situs inversus and congenital epigastric hernia [8].

Peritoneal encapsulation is usually asymptomatic; therefore, it is often diagnosed accidentally during laparotomy or autopsy. In extremely rare cases, as in the presented one, the patient may present with small bowel obstruction [1, 9]. It is, however, difficult to diagnose peritoneal encapsulation preoperatively in such cases since the radiological findings are usually normal or nonspecific [2, 7].

We did a literature review for 25 cases of peritoneal encapsulation that have been presented with a picture of acute or chronic small bowel obstruction. Their symptoms include colicky abdominal pain, nausea, anorexia, and vomiting. All of them were treated successfully by surgical resection of the sac (Table 1).

In conclusion, cases with small bowel obstruction require immediate surgery, including excision of the accessory peritoneal membrane and lysis of the adhesions between the small bowel. After surgery, the survival rate is high, and the recurrence is low [1, 10].

## 5. Conclusion

PE is a very rare congenital malformation, which, so far, remains underdiagnosed, undertreated, and mismanaged. It is more rarely associated with acute small bowel obstruction. Such patients need high clinical suspicion, should be investigated appropriately, and usually require hospitalization and emergency surgical intervention.

## Figures and Tables

**Table 1:** A summary of the characteristics of a worldwide 25 cases of peritoneal encapsulation that have been published previously.

Case	Author name	Year	Country	Age	Sex	Presentation	History of presentation	Management
1	Tojal, André, et al.	2021	Portugal	41	M	Small bowel obstruction	Colicky epigastric abdominal pain associated with bilious vomiting	Surgical resection of sac
2	Lasheen, Omar, and Mohamed ElKorety	2020	UK	41	M	Small bowel obstruction	Abdominal pain for 1 wk. associated with nausea, repeated vomiting, and relative constipation	Limited resection of the ileum with anastomosis
3	Robbins, K.J., Kooperkamp, H.Z. and Corsetti, R.L	2019	New Orleans, LA	82	M	Small bowel obstruction+Meckel diverticulum	Diffuse abdominal pain accompanied by nausea and anorexia	Surgical resection of sac
4	Renko, Abagayle E., Katelin A. Mirkin, and Amanda B	2019	USA	38	M	Small bowel obstruction	Severe, sharp, right lower Quadrant abdominal pain with abdominal distention, for 24 hours	Surgical resection of sac+adhesiolysis
5	Toma, Elena-Adelina, et al.	2019	Romania	21	M	Small bowel obstruction	Intense-abdominal pain, asymmetrical abdominal distension	Surgical resection of sac
6	McMahon, James, et al.	2018	Australia	20	M	Small bowel obstruction	Intermittent-severe abdominal pain for 7 years	Surgical resection of sac
7	Wolski, Marek, et al.	2017	Poland	12	M	Intestinal strangulation	Vomiting and abdominal pain for 2 days	Surgical resection of sac
8	Arumugam, P. K., and A. K. Dalal.	2017	India	22	F	Small bowel obstruction	Abdominal pain, vomiting, and abdominal distension	Surgical resection of sac
9	Griffith, D. G. L., M. Boal, and T. Rogers	2017	UK	12	M	Small bowel obstruction	Abdominal pain and vomiting for 1 wk.	Surgical resection of sac
10	Zoulamoglou, Menelaos, et al.	2016	Greece	28	F	Small bowel obstruction	Intermittent abdominal pain for 1 yr, asymmetric distension of the abdomen	Surgical resection of sac
11	Stewart, David, Rajay Rampersad, and Sebastian K. King	2014	Australia	16	M	Small bowel obstruction	Intermittent, chronic abdominal pain, and nonbilious vomiting, since the age of 4 years	Surgical resection of sac
12	Wani, Imtiaz, et al.	2013	India	28	M	Small bowel obstruction	Generalised, intermittent abdominal pain and bilious vomiting since 21 days	Surgical resection of sac
13	Mitrousias, Vasileios, et al.	2012	Greece	87	F	Small bowel obstruction	Bilious vomiting and abdominal pain for 3 days	Surgical resection of sac
14	Shamsuddin, Syed, et al.	2012	Pakistan	16	F	Small bowel obstruction	Abdominal pain and distension, vomiting, and weight loss for 5 days	Surgical resection.
15	Sherigar, Jagannath M., Brendon McFall, and Jaweed Wali	2007	United Kingdom	82	F	Small bowel obstruction	Lower abdominal pain, progressive abdominal distension, and vomiting for 3 days	Surgical resection of sac
16	Chew, M. H., et al.	2006	Singapore	38	M	Small bowel obstruction	Right groin pain and swelling for two months	Surgical resection of sac
17	Shioya, Takeshi, et al.	2005	Japan	34	M	Small bowel obstruction+right inguinal hernia	Colicky pain, abdominal fullness, and vomiting	Surgical resection of sac
18	Mordehai et al.	2001	Israel	15	F	Small bowel obstruction	Episodic crampy abdominal pain for 6 months	Surgical resection of sac
19	Okobia, M.N., U. Osime, and I. Evbuomwan	2001	Nigeria	15	F	Small bowel obstruction	Abdominal pain	Surgical resection of sac
20	Lee, Seong, et al.	2000	South Korea	22	F	Small bowel obstruction	Intermittent abdominal pain and distension	Surgical resection of sac
21	Casas, J. Dario, A. Mariscal, and N. Martinez	1998	Spain	43	M	Small bowel obstruction	Intermittent abdominal pain for 6 months	Surgical resection of sac
22	Adedeji, O. A., and W. A. McAdam	1994	UK	40	M	Small bowel obstruction	Constant lower abdominal pain associated with nausea, anorexia, and vomiting for days	Surgical resection of sac
23	Tsunoda, Tsukasa, et al.	1993	Japan	52	M	Small bowel obstruction+central abdominal mass	Abdominal fullness and discomfort for 1 month	Surgical resection of sac
24	Huddy, S. P. J., and M. E. Bailey.	1988	UK	56	M	Small bowel obstruction	Intermittent colicky abdominal pain	Surgical resection of sac
25	Lifschitz, O., Tiu, J. & Sumeruk, R. A	1987	Ciskei	66	M	Small bowel obstruction	Abdominal pain, distension vomiting, for 3 wk.	Surgical resection of sac

## References

[1] Sherigar J. M., McFall B., Wali J. (2007). Peritoneal encapsulation: presenting as small bowel obstruction in an elderly woman. *The Ulster Medical Journal*.

[2] Naraynsingh V., Maharaj D., Singh M., Ramdass M. J. (2001). Peritoneal encapsulation: a preoperative diagnosis is possible. *Postgraduate Medical Journal*.

[3] Zoulamoglou M., Flessas I., Zarokosta M. (2016). Congenital peritoneal encapsulation of the small intestine: a rare case report. *International Journal of Surgery Case Reports*.

[4] Renko A. E., Mirkin K. A., Cooper A. B. (2019). Peritoneal encapsulation: a rare cause of small bowel obstruction. *BMJ Case Reports*.

[5] Cleland J. (1868). On an abnormal arrangement of the peritoneum, with remarks on the development of the mesocolon. *Journal of Anatomy and Physiology*.

[6] Dave A., McMahon J., Zahid A. (2019). Congenital peritoneal encapsulation: a review and novel classification system. *World Journal of Gastroenterology*.

[7] Teixeira D., Costa V., Costa P., Alpoim C., Correia P. (2015). Congenital peritoneal encapsulation. *World Journal of Gastrointestinal Surgery*.

[8] Ince V., Dirican A., Yilmaz M., Barut B., Ersan V., Yilmaz S. (2012). Peritoneal encapsulation in a patient with incomplete situs inversus. *Case reports in surgery*.

[9] Al-Taan O. S., Evans M. D., Shami J. A. (2010). An asymptomatic case of peritoneal encapsulation: case report and review of the literature. *Cases Journal*.

[10] Rajagopal A. S., Rajagopal R. (2003). Conundrum of the cocoon. *Diseases of the Colon & Rectum*.

